# Antidiabetic Activity of Widely Used Medicinal Plants in the Sri Lankan Traditional Healthcare System: New Insight to Medicinal Flora in Sri Lanka

**DOI:** 10.1155/2021/6644004

**Published:** 2021-02-09

**Authors:** Keddagoda Gamage Piyumi Wasana, Anoja Priyadarshani Attanayake, Kamani Ayoma Perera Wijewardana Jayatilaka, Thilak Priyantha Weerarathna

**Affiliations:** ^1^Department of Biochemistry, Faculty of Medicine, University of Ruhuna, Galle, Sri Lanka; ^2^Department of Medicine, Faculty of Medicine, University of Ruhuna, Galle, Sri Lanka

## Abstract

The use of medicinal plant extracts and their isolated bioactive compounds for the management of diabetes mellitus has been tremendously increased in recent decades. The present study aimed at providing in-depth information on medicinal flora that has been widely used in the Sri Lankan traditional healthcare system for the management of diabetes mellitus. The data of this review article were obtained from published articles from January 2000 to September 2020 in scientific databases of PubMed, Web of Science, and Google Scholar. In this review, a total number of 18 medicinal plants with the antidiabetic activity were expressed, and their isolated antidiabetic active compounds were highlighted as new drug leads. Results of the reported studies revealed that medicinal plants exert a potent antidiabetic activity via both in vitro and in vivo study settings. However, bioactive compounds and antidiabetic mechanism (s) of action of many of the reported medicinal plants have not been isolated/elucidated the structure in detail, to date. Reported antidiabetic medicinal plants with other properties such as antioxidant and antihyperlipidemic activities deliver new entities for the development of antidiabetic agents with multiple therapeutic targets. This is a comprehensive review on potential antidiabetic activities of the Sri Lankan medicinal plants that have been widely used in the traditional healthcare system. The information presented here would fill the gap between the use of them by traditional healers in the traditional medicine healthcare system in Sri Lanka and their potency for development of new drug entities in future.

## 1. Background

Diabetes mellitus (DM) is an emerging health burden caused due to deficiency of production of insulin and/or resistance to insulin. According to the International Diabetes Federation (IDF) data in 2019, 463 million adults (20–79 years) have been suffered from DM, and this number is projected to reach 700 million by 2045 [1]. The South East Asia Region, the epicenter of DM, accounts for 87.9 million individuals with DM of whom 34.3% live in urban areas and 49.4% live in cities, based on the data in 2019 [1]. The majority of individuals (99.2%) live with DM in middle-income countries of the South Asia region. Sri Lanka, a developing country located in the South Asia region, has a prevalence of 8.7% in adult population (20–79 years) according to the estimates in 2019 [1].

The high degree of hyperglycemia in patients with DM associates with macrovascular complications as coronary artery disease, peripheral arterial disease, stroke, and with microvascular complications as diabetic nephropathy, neuropathy, and retinopathy [2]. The foremost risk factors for the development of diabetes associated complications include oxidative stress, inflammation, and dyslipidemia. Uncontrolled hyperglycemia in patients with DM leads to nonenzymatic glycation of proteins, glucose oxidation, and increases lipid peroxidation. All abovementioned biochemical alterations cause increase of reactive free radicals and thereby increase the oxidative stress [3]. The formation of several proinflammatory cytokines such as interleukin-6 (IL-6) and tumor necrosis factor-*α* (TNF-*α*) in diabetic patients emerge the chronic inflammation in the progression of DM and its complications. Several factors including insulin deficiency and/or resistance, production of adipocytokines, and abandoned hyperglycemia in patients with DM contribute to the alterations of lipid metabolism and thereby lead to develop dyslipidemia [4]. Accordingly, oxidative stress, inflammation, and dyslipidemia contribute to the development of diabetes associated complications. Therefore, it is important to develop antidiabetic agents targeting diabetes and its associated complications. To date, different therapeutic dilemmas are available in the management of DM and complications. The well-known classes of oral hypoglycemic agents are biguanides, sulfonylureas, meglitinides, thiazolidinediones, *α*-glucosidase inhibitors, and dipeptidyl peptidase-IV inhibitors. However, the beneficial effects of antidiabetic drug therapy are associated with some adverse effects such as hypoglycemia and weight gain [5, 6]. Despite the presence of new treatment options for type 2 diabetes mellitus (T2DM) changing markedly from 2006 to 2013, the overall glycemic control has not improved as much as expected [7]. The oral hypoglycemic agents failed to reduce the mortality rate associated with diabetes and its complications. The growing impact of diabetes mellitus and its associated complications lead to one death per every eight second globally [1, 8]. After the approval of sodium-glucose cotransporter type 2 inhibitors, as the newest antidiabetic agent to manage diabetes by FDA in 2013, new oral hypoglycemic drugs have not been introduced to date. Thus, there is an urgent need for development of safe and effective novel pharmaceutical agents and/or scientific validation of alternative therapies for the management of DM and its associated complications.

### 1.1. Medicinal Plants for Diabetes Mellitus

Long before the birth of orthodox Western medicine, medicinal flora has been used to manage wide range of diseases including diabetes mellitus. Globally, the prevalence of patients who use medicinal plants for the management of diabetes mellitus has increased in recent years. The WHO has estimated that 25% of the currently available drugs are derived from plants. Among the small-molecule drugs developed over the past 25 years or so, 5% was from natural products, 27% was derivative of natural products, and 30% was synthetic drugs inspired by natural products [9]. The study of galegine and related molecules originated from the plant *Galega officinalis* (synonym: *Galega bicolor*) in the first half of the 20^th^ century is regarded as an important milestone in the development of antidiabetic pharmacotherapy. This effort led ultimately to the discovery of metformin, currently recommended as the first line drug therapy for the management of T2DM. Accordingly, plants used in the traditional healthcare system are valuable resources of pharmacophores which could be useful for the development of novel antidiabetic agents. To date, several herbal medicines have been targeted in depth pathophysiology of diabetes mellitus through anti-inflammatory, antioxidant, antihyperlipidemic, and antihyperglycemic mechanisms [10, 11].

### 1.2. Sri Lankan Medicinal Flora and the Traditional Healthcare System

Sri Lanka is an island republic in the Indian Ocean, lying off the southeastern tip of the Indian subcontinent and located between 50 and 100N and latitude and 790 and 820EM longitude. Sri Lanka has a tropical climate with monsoons. The average monthly temperature in the island is 13°C–31°C (55°F–87°F). The country receives average precipitation of more than 3,810 mm (150 inches) to 1,270 mm (about 50 inches) of rain each year. All the geographical facts affect the high percentage of dense evergreen rain forests in Sri Lanka where an immense number of indigenous and exotic medicinal plants are grown. Sri Lankan flora is absolutely rich and diverse and has contributed to the indigenous population accumulating a vast heritage of traditional healing for number of diseases.

Four systems of traditional medicine such as Ayurveda, Siddha, Unani, and Deshiya Chikitsa have been accepted in Sri Lanka since ancient times. In Ayurveda and Deshiya Chikitsa, herbal preparations are widely used, while in Siddha, mineral preparations are used. The Unani system differs from the three traditional systems. Sri Lankan traditional practitioners are able to manage several pathogenic conditions, ailments, including DM, using herbal medicines [12]. At present, Ayurveda has become the most popular system of traditional medicine in Sri Lanka [13]. The Ayurveda system in Sri Lanka nearly uses 2000 medicinal plants [14]. Interestingly, about 60–70% of Sri Lankans use medicinal plants to manage undesirable health conditions [15]. However, the traditional healthcare system in Sri Lanka is not popular as traditional Chinese medicine and Indian Ayurveda due to lack of scientific scrutinization. The present review describes the scientific validation and /or antidiabetic potential of Sri Lankan medicinal plants that have been widely used in the traditional healthcare system for the management of DM and its associated complications with an aim of elaborating the potency of developing antidiabetic agents/drug leads.

## 2. Methodology

PubMed, Web of Science, and Google Scholar databases were searched for studies investigating antidiabetic plants for prevention and management of DM in the Sri Lankan traditional healthcare system up to September 2020 from January 2000. Furthermore, studies carried out based on the isolation of antidiabetic active compounds from the investigated plants were gathered. Additional information were collected from the reference lists of articles. The title, abstract, and references of articles were examined by two independent reviewers. The information on plants, trial duration, sample size, dose of target plant material or extract, output, and side effects were independently examined.

## 3. Results and Discussion

A total number of eighteen medicinal plants are deeply reviewed as mentioned below. The following expressed studies related to the antidiabetic activity have been carried out on medicinal flora from Sri Lankan origin.

### 3.1. *Salacia reticulata* var. b-diandra Weight

*Salacia reticulata* var. b-diandra Weight (Family, Celestraceae) is a woody climbing shrub which is indigenous to Sri Lanka and India. The climber *S. reticulata* is widely available in low country rain forests in the Southern region of Sri Lanka. *S. reticulata* is commonly named as kotala himbatu or himbutu-wel in Sinhalese. Ayurvedic practitioners recommend this climber in the management of DM. In addition to the use of the plant in the treatment of DM, decoctions of *S. reticulata* have been used for thousands of years to treat asthma, itching, gonorrhea, skin diseases, and hemorrhoids. *S. reticulata* is used as a supplementary food by Japanese to prevent DM and obesity. In an acute study, different fractions of *S. reticulata* (root and stem) such as petroleum ether (233 mg/kg), ethyl acetate (29 mg/kg), methanol (350 mg/kg), and water (500 mg/kg) were administrated to alloxan-induced diabetic rats (150 mg/kg) [16]. The group treated with methanol fraction showed a significant reduction of blood glucose concentration when compared to the control group. A long-term (120 days) study on methanol extract of *S. reticulata* (175 mg/kg) also revealed the antihyperglycemic activity of *S. reticulata* in the same animal model [16]. The antihyperglycemic potential was evaluated through a reduction of glycated hemoglobin (HbA_1C_), fasting blood glucose concentration, fructosamine, and an increase in the concentration of serum insulin [16]. A double-blind randomized placebo controlled crossover study using *S. reticulata* of Sri Lankan origin reported that there was a reduction in fasting blood glucose concentration in T2DM patients who were treated with the herbal preparation of *S. reticulata* [17]. The hypoglycemic effect was confirmed using the results of HbA_1C,_ fasting blood glucose concentration upon the completion of the study in six months. Furthermore, the study indicated a beneficial effect of the adorn action of *S. reticulata* with an oral hypoglycemic drug, glibenclamide. Several antidiabetic compounds such as salacinol, kotalanol, ponkorinol, salaprinol, and their corresponding de‐O‐sulfonated compounds were identified from *S. reticulata* [18]. Among those molecules, salacinol is well documented as a potent natural *α*-glucosidase [19]. Thus, the reported antihyperglycemic activity of *S. reticulata* could be partially due to intestinal *α*-glucosidase inhibitory activity. Moreover, inhibition of *α*-glucosidase enzyme delays the glucose absorption into blood and suppresses postprandial hyperglycemia resulting in improvement of the blood glucose level [19–22].

### 3.2. *Trichosanthes cucumerina* (L.)

*Trichosanthes cucumerina* (L.) (Family, Cucurbitaceae) is widely available in Asian countries [23]. *T. cucumerina* plant is known as dummella in Sinhala and kattuppeyppudal, pudaj, and pcyppudal in Tamil [23]. Every part (roots, leaves, fruits, and seeds) of this plant possesses a medicinal value. The root is used for bronchitis, headache, and boils. The leaves are important to relieve liver congestion in patients with liver diseases. The decoction of the aerial parts of *T. cucumerina* is used for bilious fevers, boils, sores, skin eruptions, and DM [22, 24–26]. The aerial parts of *T. cucumerina* are widely recommended in the management of DM by Ayurvedic practitioners in Sri Lanka [22, 24, 25]. The hot water extract of aerial parts of *T. cucumerina* exerted the hypoglycemic activity in streptozotocin-induced diabetic rats. A dose-dependent reduction in blood glucose concentration was observed at the 750 mg/kg dose on the oral glucose tolerance test [27]. Even though hot water extract of aerial parts of *T. cucumerina* did not show immediate reduction in fasting blood glucose concentration in streptozotocin-induced diabetic rats, the long-term study showed a gradual reduction on the 14^th^ day (56.8%) and 28^th^ day (64.4%). Therefore, the antihyperglycemic activity of the hot water extract of aerial parts of *T. cucumerina* might occur through induction of *β*-cells, direct increment of peripheral glucose utilization, and lack of intestinal glucose absorption [28, 29]. Phenolic compounds (flavonoids and tannins) present in *T. cucumerina* attribute to the antidiabetic potential as suggested by several authors [30–33]. A number of antidiabetic/antioxidant compounds (carotenoids, flavonols, and flavanones) have also been identified in *T. cucumerina* in Sri Lankan species [34]. However, a complete structure elucidation of antidiabetic compounds has not been reported to date.

### 3.3. *Gymnema lactiferum* (L.) R. Br. ex Schult

*Gymnema lactiferum* (L.) R. Br. ex Schult. (Family, Apocynaceae) is a green leafy vegetable which is available in the wet zone of Sri Lanka [35]. This plant is called as Kuringnan, Muva kiri-vel, or Masbedda in Sinhala. *G. lactiferum* is popular as an antidiabetic plant and is used in different preparations as gruels, salads, and curry. Preclinical and clinical studies based on *G. lactiferum* reported its hypoglycemic activity [36–38]. A study carried out with the administration of *G*. *lactiferum* aqueous leaves extract (1.25 g/kg) for 28 days showed a reduction in blood glucose concentration in streptozotocin-induced T2DM rats [36]. The factors responsible for the glucose lowering effect might be due to the triggering insulin activity and inhibiting the intestinal glucose absorption as suggested by Thushari et al. [36]. A clinical trial carried out with the ingestion of a suspension of *G. lactiferum* leaves powder to T2DM patients for four weeks was able to demonstrate gradual reduction in fasting blood glucose concentration [37]. The results of the study clearly indicated that the freeze-dried powder of the aqueous leaf extract of *G. lactiferum* was able to overwhelm postprandial hyperglycemia [37]. Another scientific study revealed that aqueous leaf extract of *G. lactiferum* has *α*-amylase and *α*-glucosidase inhibitory potential [38]. The antidiabetic phytoconstituents have not been isolated in *G. lactiferum* species grown in Sri Lanka. It is reported that *G. lactiferum* is evidently a form of *G. sylvestre*. Similar to *G. lactiferum*, leaves of *G. sylvestre* are widely used in the treatment of several ailments including diabetes mellitus, and this fact is further strengthened by the isolated antidiabetic components, gymnemic acids (gymnemic acid I, II, III, and IV), from *G. sylvestre* [39–41].

### 3.4. *Coccinia grandis* (L.) Voigt

*Coccinia grandis* (L.) Voigt (Family, Cucurbitaceae) is widely distributed in Sri Lanka and is named as Kem wel or kowakka in Sinhala, kovai in Tamil, and scarlet gourd in English. Every part of this plant is used for different purposes [42]. *C. grandis* is very popular in traditional medicine as an antidiabetic agent [43–46]. The hot water extract of *C. grandis* leaves showed an improvement of glucose tolerance (8.50%) in healthy rats after the oral administration of the plant extract at a single dose (0.75 g/kg) experiment. Accordingly, a dose-dependent improvement of glucose tolerance was also observed in streptozotocin-induced diabetic rats (*p* < 0.05) [45]. A study carried out with the aqueous extract of *C. grandis* leaves in streptozotocin-induced diabetic rats for 30 days demonstrated a significant antihyperglycemic activity at its optimum therapeutic dose of 0.75 g/kg [47]. The serum glycated hemoglobin and serum concentration of fructosamine were reduced by 33% and 34%, respectively, while an increment in serum insulin and C-peptide concentration was observed by 74% and 53%, respectively, in streptozotocin-induced diabetic rats. Results confirmed that the aqueous extract of *C. grandis* leaves possesses the antihyperglycemic activity via the increased synthesis of insulin in regeneration of *β*-cells [47, 48]. In addition, the water extract of *C. grandis* leaves at the dose of 0.75 g/kg showed significant antihyperlipidemic and antioxidative activities in streptozotocin-induced diabetic rats [49]. The phytochemical screening carried out on aqueous extract of *C. grandis* leaves revealed the presence of several compounds as polyphenols, alkaloids, flavonoids, and saponins [50]. A few numbers of compounds such as cucurbitacins B and D, cephalandrol, cephalandrin A and B, and related analogs have been identified as bioactive compounds responsible for the glucose lowering effect in the standardized extract of *C. grandis* aerial parts [51]. A clinical trial based on herbal dietary meal consisting *C. grandis* leaves showed the glucose lowering effect in healthy individuals [52]. The hypoglycemic potential could be due to decreased glucose absorption from the gut, increment of insulin synthesis, reduction of glucose release from the liver, and increment of glucose uptake by adipocytes and muscle cells as reported by Munasinghe et al. [52].

### 3.5. *Syzygium cumini* (L.) Skeels

*Syzygium cumini* Linn Skeels (Family, Myrtaceae) is a tropical tree which is known as madan in Sinhala. Madan is a very popular antidiabetic plant in Ayurveda and in Indian folk medicine. A decoction of *S. cumini* bark has been widely recommended in the management of DM and its associated complications by Sri Lankan Ayurvedic physicians since ancient era. *S. cumini* was the most cited species by Siddha healers in the Eastern province, Sri Lanka, as an active agent in the antidiabetic preparations [53]. A significant blood glucose reduction was observed in healthy mice treated with the methanol extract of *S. cumini* bark (0.25 mg/g) [54]. Hexane, ethyl acetate, methanol, and water extracts resulted from sequential extraction on leaf powder of *S. cumini* at a dose of 2 mg/mL showed an inhibition of fructosamine formation, protein glycation, and protein cross-linking [55]. Moreover, the same study revealed that the water extract exerts the highest *α*-glucosidase inhibitory activity with an IC_50_ value of 0.69 *µ*g/mL. Several antidiabetic compounds as gallic acid, umbelliferone, and ellagic acid were identified in the aqueous extract of *S. cumini* bark [56]. The antioxidant activity of the water extract of *S. cumini* was determined using 2, 2-diphenyl-1-picryl-hydrazyl-hydrate (DPPH), ferric reducing antioxidant power (FRAP), and nitric oxide (NO) assays [57]. The ursolic acid and oleanolic acid were identified as main antioxidant/antidiabetic compounds from the leaves of *S. cumini* [58]. The isolated compounds as gallic acid, umbelliferone acid, ellagic acid, ursolic acid, and oleanolic acid were found to demonstrate antidiabetic, antioxidant, antilipidemic, and cardioprotective properties [58].

### 3.6. *Zingiber officinale* Roscoe

*Zingiber officinale* Roscoe (Family, Zingiberaceae) is called as ginger in English and inguru in Sinhala. The rhizome of ginger is recommended in the traditional medicine for the treatment of DM, hypertension, arthritis, toothache, asthma, and several infectious diseases. Owing to these medicinal values, *Z. officinale* is used as an active ingredient in well-liked Sri Lankan product of “Paspanguwa.” Several studies showed that ginger enhances the insulin sensitivity and thereby beneficial in the management of DM [59–61]. In addition to the antidiabetic activity, *Z. officinale* exerts anti-inflammatory, hypolipidemic, and hypocholesterolemic activities [62]. *Z. officinale* plant is rich in phenolic compounds. The major chemical constituents isolated from ginger are pungent vanilloids, gingerol, paradol, shogaols, and zingerone. A study has shown that the aqueous extract of ginger (peeled 10 g of ginger in 100 ml of distilled water) exerted potent glucosidase and amylase inhibitory activities [63]. It was evident through the 10% significant reduction of glucose concentration in cooked rice (5 g of rice in 25 mL of distilled water) treated with *α*-glucosidase (50 mg in 27 mL of distilled water) and ginger extract (2 mL). A significant reduction of glucose concentration as 15% was observed upon the administration of ginger extract and cooked rice. Interestingly, the results were comparable to the cooked rice treated with the enzyme and the reference compound acarbose [63]. However, detailed mechanisms of action (s) of *Z. officinale* in vivo, isolation of antidiabetic compounds, and elucidation of structures have not been reported to date.

### 3.7. *Canthium coromandelicum* (L.)

*Canthium coromandelicum* (L.) (Family, Burm) is available in dry scrub and monsoon forests in Sri Lanka. It is called as kara in Sinhala. *C. coromandelicum* exerts antimicrobial, antioxidant, hepatoprotective, and antibacterial properties [64]. According to folklore, the leaves of *C. coromandelicum* are used in the preparation of dry curry (mallum). Indigenous practitioners have recommended the leaves of this plant to be included in diet of patients with DM. Scientific investigations carried out on single and multiple doses of the water extracts of *C. coromandelicum* leaves in Wistar rats demonstrated a potent hypoglycemic activity [65]. It was demonstrated by a decrease in serum glucose concentration in the range of 15.4%–25.7% at the selected dose range of 15–30 g/kg. Furthermore, a study suggested that the reduction or inhibition of glucose absorption by the fiber and pectin in the leaves extract of *C. coromandelicum*. This was evident through a reduction in fasting and/or the postprandial serum glucose concentration on the 8^th^ and 15^th^ day by administration of *C. coromandelicum* leaves decoction (20 g/kg). The antidiabetic phytoconstituents as glycosides, tannins, flavonoids, and disaccharide were identified [64, 66]. However, elucidation of the structures of antidiabetic active compounds of *C. coromandelicum* from Sri Lanka origin has not been performed to date.

### 3.8. *Ipomoea aquatica* Forsk

*Ipomoea aquatica* Forsk (Family, Convolvulaceae) is known as kankun in Sinhala and koilangu or sarkareivalli in Tamil. The plant is widely available in marshy areas [43] and is used for the treatment of disorders including jaundice, nerves debility, high blood pressure, and DM in Ayurveda. A study carried out using the edible portion of the water extract of *I*. *aquatica* in healthy, male Wistar rats demonstrated a hypoglycemic effect. A significant percentage of reduction in the serum glucose concentration by 33.6% at a single dose (3 g/kg) and a 25% reduction at multiple doses (3.4 g/kg for 7 days) were observed in the study [67]. The secretion of insulin and reduction of glucose absorption were noted as antidiabetic mechanisms [67]. The treatment of fresh, edible portion of *I. aquatica* (3.4 g/kg for seven days) for streptozotocin-induced diabetic Wistar rats showed significant reduction (48.6%) of fasting serum glucose concentration [68]. Administration of the aqueous extract of the edible portion of *I. aquatica* (100 g of fresh edible portion of *I. aquatica* blend with boiled water) in patients with newly diagnosed T2DM was able to significantly reduce serum glucose concentration by 29.4% at 2 hours after glucose load [68]. There are no published reports on the isolation and structure elucidation of antidiabetic compounds from Sri Lankan *I. aquatica* species to date.

### 3.9. *Aegle marmelos* (L.) Correa

*Aegle marmelos* (L.) Correa (Family; Rutaceae) is commonly known as bael fruit tree in English and available in Asian countries. Every part of the plant (root, leaf, trunk, and fruits) is used in the management of wide variety of disorders as dysentery, piles, dyspepsia, jaundice, scrofula, indigestion, and chronic fever in traditional medicine [43]. Based on the scientific investigations, the water extract of dry flower of *A. marmelos* exerted potent hypoglycemic and antihyperglycemic activities [69]. A reduction in serum glucose concentration as 15% and 21% was observed in healthy and alloxan-induced diabetic rats at a single dose experiment (200 mg/kg), respectively. Furthermore, a study in healthy rats indicated that the serum glucose concentration was reduced by 29%, and the results were comparable to the drug action of metformin (27%) and glibenclamide (26%). A 38.5% reduction in glucose concentration was observed two hours after glucose load in multiple dose experiments (500 mg/kg for 7 days). Several antidiabetic compounds such as coumarins, alkaloids, and tannins were isolated from the plant of *A. marmelos* [70]. However, complete structure elucidation of antidiabetic compounds and antidiabetic mechanisms have not been reported for *A. marmelos* to date.

### 3.10. *Alpinia calcarata* Roscoe

Rhizomes of *Alpinia calcarata* Roscoe (Family, Zingiberaceae) is known as Heen araththa or Katu kiriya in Sinhala and snap ginger in English. *A. calcarata* is widely available in Asian countries including Sri Lanka. Ayurvedic practitioners recommended *A. calcarata* in the management of rheumatism, pain, catarrh, anorexia, excessive sweating, and hoarseness of voice. Various studies demonstrated that *A. calcarata* possessed antibacterial, antifungal, antioxidant, and gastroprotective activities [71–74]. Scientific investigations carried out with the oral administration of the hot water and hot ethanolic extract of rhizomes of *A. calcarata* to normoglycemic and streptozotocin-induced diabetic rats demonstrated hypoglycemic and antihyperglycemic effects, respectively [74]. The administration of range of doses in the hot water decoction (250–1000 mg/kg) and hot ethanol extract (250–1000 mg/kg) significantly dropped the fasting blood glucose concentration up to six hours in normoglycemic rats. A dose of 500 mg/kg showed the maximum hypoglycemic activity after two hours of administration (34% reduction for hot water decoction and 40% reduction for ethanolic extract). The hypoglycemic effect of the hot water extract of *A. calcarata* at 500, 750, and 1000 mg/kg was comparable to the effect of tolbutamide in diabetic rats. Both hot water and hot ethanol extracts significantly improved the glucose tolerance within the three-hour period. *A. calcarata* extract exerted a potent antidiabetic activity via inhibition of intestinal absorption of glucose. Phytochemical screening of hot water and ethanol extracts of rhizomes of *A. calcarata* revealed the presence of alkaloids, polyphenols, flavonoids, steroids, saponins, and tannins. Phenolic compounds such as flavonoids and tannins are suggested to be responsible for the reported antidiabetic activity [75, 76]. However, the exact antidiabetic compounds of *A. calcarata* have not been identified to date. Polyphenols present in the extract exerted a significant antioxidant activity [77], and the antioxidant activity of cold ethanol extract of *A. calcarata* rhizomes was proven using the DPPH assay [73].

### 3.11. *Cinnamomum zeylanicum* Blume

*Cinnamomum zeylanicum* Blume (Family, Lauraceae) is called as Ceylon cinnamon or true cinnamon in English and kurundu in Sinhala. The plant is indigenous to Sri Lanka. This plant is cultivated in the moist low areas in Sri Lanka. Cinnamon bark is used as an antiemetic, antidiarrheal, antiflatulent, and as a general stimulant in Ayurveda [73, 78]. A scientific investigation carried out on freeze-dried form of the hot water decoction of *C. zeylanicum* in normal and streptozotocin-induced diabetic rats demonstrated a blood glucose lowering effect [79]. In this study, short-term and long-term effects of freeze-dried form of hot water decoction of *C. zeylanicum* was investigated by administration at a dose of 600 mg/kg. The polyphenols present in *C. zeylanicum* attributed to its antidiabetic activity [80]. The main organic compound present in cinnamon is the cinnamic aldehyde (cinnamon oil). Beside the oil, other bioactive ingredients such as sugar, mannite, starch, mucilage, and tannic acid are available in the bark. Eugenol is the main constituent present in the oil from leaves, while camphor, eucalyptol, and safrol are present in the oil from roots of cinnamon. In addition, a negligible amount of coumarin content is present in *C. zeylanicum* [43]. Furthermore, freeze-dried hot water extract of stem bark of *C. zeylanicum* was subjected to determine its antidiabetic activity via randomized, double-blind, placebo controlled clinical trial carried out for a period of four months in patients with DM [81]. However, the outcomes of this clinical trial are unavailable to date.

### 3.12. *Piper betle* (L.)

*Piper betle* (L.) (Family, Piperaceae) is a semiwoody climber widely available in Sri Lanka [43]. It is known as bulath or nagawalli in Sinhala and beetle in English. *P. betle* is used in the management of fever, night blindness, infertility, ulcers, and hypertension. Studies showed that the *P. betle* leaves possessed antimicrobial, gastroprotective, wound healing, hepatoprotective, and antioxidant properties [82–85]. Administration of freeze-dried powder of the hot water extract of *P. betle* leaves (200 mg/kg) and cold ethanolic extract of *P. betle* leaves (200 mg/kg) to adult cross-bred male albino rats showed 14, 11, and 10% and 16, 12, and 11% reduction in serum glucose concentration at 1^st^, 2^nd^, and 3^rd^ hours, respectively [86]. The reference hypoglycemic drug, tolbutamide, also improved the glucose tolerance up to 3 hours, and the impairment was comparable to that of the *P. betle* extract [86]. Freeze-dried powder of the hot water extract of *P. betle* leaves (200 mg/kg) was able to significantly reduce serum glucose concentration in streptozotocin-induced diabetic albino rats at the 2^nd^ and 4^th^ hour of the posttreatment [86]. In addition, cold ethanol extract of the *P. betle* leaves showed potent in vitro antioxidant activity in terms of the DPPH scavenging activity and thiobarbituric acid reactive substances (TBARS) [86]. Moreover, the same study reported that administration of hot water extract of *P. betle* leaves (200 mg/kg) for 42 consecutive days in adult cross-bred male albino rats was able to significantly increase (*p* < 0.05) the glycogen content in skeletal muscle and liver by 44% and 105%, respectively. Thus, it was important to highlight that the antidiabetic activity of the hot water extract of *P. betle* leaves due to accumulation of glycogen in the liver and skeletal muscle. This increased glycogenesis may result from enhancement of uptake of glucose from the liver and from the skeletal muscle by sensitization of insulin receptors and/or induction of the activity of enzymes involved in glycogen synthesis [86]. Preliminary screening carried out on Sri Lankan *P. betle* leaves revealed the presence of several phytochemicals as polyphenols, alkaloids, and steroids [87, 88]. However, structure elucidation of antidiabetic compounds has not been reported to date.

### 3.13. *Spondias pinnata* (L. f.) Kurz

*Spondias pinnata* (Family, Anacardiaceae) is named as emberella in Sinhala, bile tree, Indian hog plum, wild mango, or traveller's delight in English, and ambalam in Tamil. This plant is widely distributed in Sri Lanka. The bark of *S. pinnata* is used in the treatment of dysentery, and juice of the leaves is used for earache. The hot water decoction of the bark of *S. pinnata* exerted dose-dependent acute hypoglycemic and antihyperglycemic activities [45, 89]. An improvement of 8.14% on glucose tolerance was observed in healthy Wistar albino rats after an oral administration of bark decoction of *S. pinnata* at the dose of 1.00 g/kg [45]. The hot water extract of *S. pinnata* bark showed a significant percentage improvement on glucose tolerance by 29.98% at the dose of 1.00 g/kg in streptozotocin-induced diabetic Wistar albino rats [45]. The water extract of *S. pinnata* bark at the dose of 1.00 g/kg showed an acute antihyperglycemic activity on glucose tolerance in alloxan-induced diabetic rats [89]. The water extract of *S. pinnata* bark (1 g/kg for 30 days) showed a long-term antidiabetic activity via the insulinotropic effect in streptozotocin-induced diabetic rats [90]. The remarkable long-term antidiabetic activity was evident through a significant reduction (*p* < 0.05) in fasting blood glucose concentration, percentage of glycated hemoglobin, and serum concentration of fructosamine as 37, 25, and 26%, respectively. In addition, the study confirmed that the extract induced the *β*-cell restoration and led to an increment in serum insulin concentration in streptozotocin-induced diabetic rats. The study also revealed that the extract (1.0 g/kg) possessed the antihyperlipidemic activity in the same model of diabetes [90]. Phytochemical screening on hot water extract of bark of *S. pinnata* revealed the presence of alkaloids, phenols, flavonoids, phytosterols, saponins, tannins, and reducing sugars [50]. A moderate total antioxidant activity was also found in the aqueous extract of *S. pinnata* bark based on DPPH, FRAP, and NO inhibition assays [50]. This was further supported with the findings of Pari and Venkateswaran [91]. However, the exact antidiabetic compounds were not reported to date.

### 3.14. *Gmelina arborea* Roxb

*Gmelina arborea* (Family, Verbenaceae) is found in Southeast Asia and in tropical Australia. It is called as Eth demata in Sinhala and beech wood in English. The plant parts are used as a demulcent, stomachic, bitter tonic, refrigerant, and laxative in Ayurveda [92]. An improvement of 7.69% on glucose tolerance was observed in healthy Wistar albino rats after oral administration of the hot water extract of *G. arborea* bark at the dose of 1.00 g/kg [45]. The hot water extract of *G. arborea* bark showed a significant (*p* < 0.05) percentage improvement on glucose tolerance by 18.42% at the dose of 1.00 g/kg in streptozotocin-induced diabetic Wistar albino rats [45]. The administration of aqueous extract of *G. arborea* bark (at the optimum dose of 1.00 g/kg) for 30 days showed the antihyperglycemic activity in streptozotocin-induced diabetic rats [93]. The antihyperglycemic activity was evident through a significant reduction (*p* < 0.05) in fasting blood glucose concentration, percentage of glycated hemoglobin, and serum concentration of fructosamine as 37%, 31%, and 28%, respectively. The same study also showed the significant improvement (*p* < 0.05) of serum insulin and *C*-peptide as 57% and 39%, respectively, in streptozotocin-induced diabetic rats upon the treatment of aqueous extract of *G. arborea* bark at the dose of 1.00 g/kg for 30 days [93]. An increased production of insulin might be responsible for its antidiabetic mechanism in vivo [93]. In addition, the antihyperlipidemic activity was also proven for the decoction of *G. arborea* bark (1.00 g/kg for 30 days) in streptozotocin-induced rats based on the changes in the lipid profile parameters [93]. The in vitro antioxidant activity of the decoction of *G. arborea* bark and methanol extracts of stem bark of *G. arborea* were investigated using DPPH, FRAP, and NO inhibition assays [94]. Phytochemical screening of hot water extract of *G. arborea* leaves suggested the presence of several compounds as alkaloids, carbohydrate, glycoside, and protein [50]. However, the exact antidiabetic compounds have not been isolated from the Sri Lankan species to date.

### 3.15. *Scoparia dulcis* (L.)

*Scoparia dulcis* (L.) (Family, Scrophulariaceae) is grown in the torrid zone. It is known as Wal koththamalli in Sinhala and licorice weed, goat weed, scoparia weed, or sweet broom in English. *S. dulcis* is traditionally used for disorders such as DM, hypertension, and bronchitis [95]. *S. dulcis* possess antimicrobial, antiulcer, antioxidant, anti-inflammatory, hepatoprotective, and antidiabetic activities [96, 97]. An improvement of 7.43% on glucose tolerance was observed in healthy Wistar albino rats after an oral administration of the hot water extract of *S. dulcis* aerial parts at the dose of 1.00 g/kg [45]. The hot water extract of *S. dulcis* aerial parts showed a significant (*p* < 0.05) percentage improvement on glucose tolerance by 25.42% at the dose of 1.00 g/kg in streptozotocin-induced Wistar albino rats induced with diabetes mellitus [45]. The antidiabetic mechanisms might be due to the inhibition of increased synthesis of insulin from pancreatic *β*-cells as suggested by Attanayake et al. [90]. Furthermore, certain responsible chemical compounds for the blood sugar lowering activity were isolated as scoparic acid A, scoparic acid D, scutellarein, apigenin, luteolin, coixol, and glutinol [96, 98]. However, the exact antidiabetic compounds of Sri Lankan species of *S. dulcis* have not been isolated to date.

### 3.16. *Adenanthera pavonina* (L.)

*Adenanthera pavonina* (L.) (Family, Fabaceae) is known as madatiya in Sinhala and Circassian bean in English. *A. pavonina* is widely distributed in Asian countries including Sri Lanka. From the immemorial time, *A. pavonina* is very renowned among Sri Lankans as a medicinal plant for the management of DM, diarrhea, dysentery, and snake bites. Seeds are used as poultice, and powered seeds are externally applied for hastening suppuration. Decoction of the bark is well known as a remedy for chronic rheumatism, gout, and intestinal hemorrhage [99]. Based on the scientific in vitro and in vivo investigations, the water extract of *A. pavonina* exerted antihyperglycemic and hypoglycemic activities. A study carried out with the oral administration of hot water extract of mature leaves of *A. pavonina* (different doses of 500, 750, and 1000 mg/kg) to normoglycemic Sprague–Dawley rats showed a significant hypoglycemic effect at the three doses up to 2 hours [99]. The methanol extract of *A. pavonina* leaves exerted a remarkable *α*-amylase inhibitory activity similar to that of reference drug acarbose [100]. The study further revealed that ethyl acetate fraction of *A. pavonina* leaves exert an antioxidant activity in terms of the DPPH free radical scavenging activity and Folin Ciocalteu's reagent [100]. Phytochemical screening performed on the plant revealed the presence of alkaloids, steroids, glycosides, polysaccharides, fatty acids, saponins, and various amino acids [101, 102]. The antidiabetic compound of oleanolic acid was isolated from the leaves of *A. pavonina* and is reported to exert insulin secretory effects in the pancreas [99].

### 3.17. *Osbeckia octandra*

*Osbeckia octandra* (Family, Melastomataceae) is one of the endemic plants in Sri Lanka. *O. octandra* is called as Heen bovitiya in Sinhala. *O. octandra* is recommended to use in the management of DM, hepatitis, and hemorrhoids by Ayurvedic practitioners in Sri Lanka [23]. A porridge prepared from young leaves of *O. octandra* is prescribed as a liver tonic [23]. Blood sugar lowering effect of *O. octandra* leaves was scientifically proven in patients with DM [103]. Supplementation of *O. octandra* leaves (dipping two spoonsful of powdered leaves in 30 mL of warm water) twice a day for 30 days led to decrease in the fasting blood glucose level [103]. A previously performed study reported the high antioxidant capacity of the decoction of *O. octandra* leaves in terms of the DPPH scavenging activity and 2, 2′-azinobis (3-ethylbenzothiazoline-6-sulphonic acid) (ABTS) diammonium salt radical cation decolorization assay [104]. In addition, the antiglycation activity of the decoction of *O. octandra* leaves was proven using bovine serum albumin assay [104]. Further studies are wanted to identify antidiabetic active compounds and the mechanisms of action in *O. octandra* leaves.

### 3.18. *Passiflora suberosa* L

*Passiflora suberosa* (Family, Passifloraceae) is commonly known as wild passion fruit, devil's pumpkin, or indigo berry. Leaves of *P. suberosa* is used in Sri Lankan traditional medicine for the treatment of diabetes mellitus and hypertension [105]. Hypoglycemic potential of *P. suberosa* leaves has been scientifically proven in male mice. The administration of different acute doses (0, 25, 50, 100, and 200 mg/kg) of aqueous extract of *P. suberosa* leaves showed significant glucose lowering potential (10%, 20%, and 24%, respectively, at the 1^st^, 3^rd^, and 5^th^ hour) in mice treated with 50 mg/kg dose [106]. Following the treatment of aqueous leaf extract of *P. suberosa* (50 mg/kg) for 30 consecutive days in male ICR (imprinting control region) mice showed significant glucose absorption (79%) from the lumen of the intestine [106]. Furthermore, significant increment of glycogen content in the liver (61%) and skeletal muscles (57%) was observed when compared to the control group in the same animal model. In parallel to blood glucose lowering potential, it was found that the leaves extract is possible to decrease the cholesterol level significantly upon the treatment of aqueous leaves extract of *P. suberosa* (50 mg/kg) for 30 days [106]. The aqueous extract of *P. suberosa* leaves showed the potent antioxidant activity in terms of DPPH scavenging assay [107]. Phytoconstituents such as alkaloids, sterols, triterpenoids, saponins, flavonoids, and tannins were identified from the aqueous extract of *P. suberosa* leaves [106]. The antidiabetic compounds have not been isolated from the leaves of *P. suberosa*.

## 4. Conclusion

**Figure 1:**
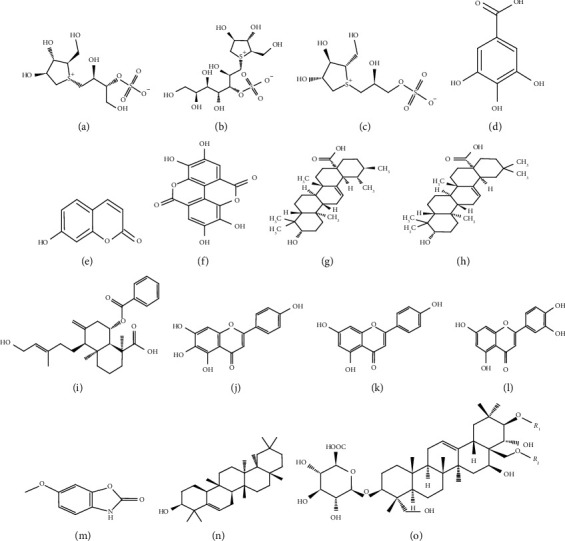
Isolated antidiabetic compounds. (a) Salacinol, (b) kotalanol, (c) salaprinol, (d) gallic acid, (e) umbelliferone, (f) ellagic acid, (g) ursolic acid, (h) oleanolic acid, (i) scoparic acid A (j) scutellarein, (k) apigenin, (l) luteolin, (m) coixol, (n) glutinol, and (o) gymnemic acid I: *R*_1_ = tigloyl, *R*_2_ = acetyl; gymnemic acid II: *R*_1_ = 2-methylbutanoyl, *R*_2_ = acetyl; gymnemic acid III: *R*_1_ = 2-methylbutanoyl, *R*_2_ = *H*; gymnemic acid IV: *R*_1_ = tigloyl, *R*_2_ = *H*.

An extensive literature survey was performed on medicinal plants, *Salacia reticulata, Trichosanthes cucumerina, Gymnema lactiferum, Coccinia grandis, Syzygium cumini, Zingiber officinale, Canthium coromandelicum, Ipomoea aquatica, Aegle marmelos, Alpinia calcarata, Cinnamomum zeylanicum, Piper betle, Spondias pinnata, Gmelina arborea, Scoparia dulcis, Adenanthera pavonina, Osbeckia octandra*, and *Passiflora suberosa*. For preclinical in vivo studies, alloxan- and streptozotocin-induced diabetic rats/mice were commonly used as the model to assess the antidiabetic activity. *α*-amylase and *α*-glucosidase inhibition assays were used to screen the antidiabetic activity in vitro. Diverse mechanisms of action are suggested for the antidiabetic activity of plant extracts. Some of the antidiabetic mechanisms are increment of peripheral utilization of glucose, increment of synthesis of hepatic glycogen, decrement of glycogenolysis, and inhibition of glucose absorption. Some plant extracts were able to improve the insulin sensitivity and pancreatic *β*-cell function. The antidiabetic effect of the reported medicinal plant extracts is attributed mostly to the mixture of phytochemicals. Plant secondary metabolites responsible for the antidiabetic activity are alkaloids, sterols, triterpenoids, saponins, flavonoids, tannins, glycosides, and disaccharide compounds. Antidiabetic active compounds (Figure 1) such as salacinol, kotalanol, salaprinol, gallic acid, umbelliferone, ellagic acid, ursolic acid, oleanolic acid, scoparic acid A, scutellarein, apigenin, luteolin, coixol, glutinol, and gymnemic acids were isolated from the reported medicinal plants. Moreover, it is worth noting that medicinal plants such as *Salacia reticulata, Coccinia grandis, Ipomoea aquatica*, and *Osbeckia octandra* have been subjected to clinical trials. Most of the methods used for clinical trials were not appropriately designed and hence led to inconclusive findings. Thus, a lot more plants have to be efficiently explored and proved clinically for the antidiabetic activity. In addition to antidiabetic activity, reported medicinal plant extracts exerted antioxidant activity in terms of DPPH, NO, FRAP, TBARS, FCR, and ABTS diammonium salt radical cation decolorization assays. In parallel to antidiabetic and antioxidant activities, the reported medicinal plant extracts showed an improvement of lipid profile parameters. Thus, it showed that these plant extracts could be used to treat secondary complications of DM and risk factors. However, further studies are warranted to investigate underline in-depth mechanisms of action toward the management of DM, associated complications, and to isolate antidiabetic active compounds. All highlighted studies in the present review have been carried out on the medicinal flora of Sri Lankan origin. Collection of the preclinical and clinical evidence by means of the antidiabetic activity on these reported medicinal flora from different origins other than Sri Lanka further strengthen the outcome of the present review. As future perspectives, the medicinal flora described in the present review might be important in the process of development of novel drug entities for DM.

## Data Availability

No data were used to support this study.
